# Galactics

**Published:** 1861-04

**Authors:** 


GALACTICS.—*Liquid food*, including milk, good nutritious soups, ale or beer, and other malt liquors. Lager-beer is much inferior to ordinary ale in promoting the secretion of milk, and principally as it would seem from the effect of the pitch with which it is impregnated, and which acts so powerfully and immediately upon the skin and kidneys, creating such a secretion of perspiration and urine, that the tendency to the breast is thus neutralized. "*Spirituous* liquors, instead of increasing, as many suppose, diminish the quantity of milk secreted."

*Fœniculum.*—Hippocrates, as well as Galen, already speaks of fennel as a means of increasing the lacteal secretion. Dioscorides ascribes the same powers to it (lib. III. chap. lxvii et seq.). According to Mitscherlich, also, it increases besides other secretions, certainly that of milk (Stille's Therap. and Materia Medica, vol. 1, p. 592). In Germany, especially, it has been tried extensively and lauded correspondingly. It is given either alone as infusion ad libit, or combined with various other articles still to enhance its power. Among the most celebrated and valuable formula is that of Hufeland:

℞. Sem. Fœniculi, ................ ʒ i;

Flav. Cort. Aurant. ............ ʒ ss;

Subcarb Magnes. ............... ʒ iij;

Sacch. Alb. .................... ʒ ij. M. ft. pulv.

Dose a teaspoonful three times a day.

I have obtained surprising results from Hufeland's formula, which I have employed in several cases, in one where the secretion had been suppressed for three weeks.—*Prof. Gardner*—*Amer. Med. Times.*

